# Glomerular spatial transcriptomics of IgA nephropathy according to the presence of mesangial proliferation

**DOI:** 10.1038/s41598-024-52581-8

**Published:** 2024-01-26

**Authors:** Sehoon Park, Minji Kang, Yong Chul Kim, Dong Ki Kim, Kook-Hwan Oh, Kwon Wook Joo, Yon Su Kim, Hyun Je Kim, Kyung Chul Moon, Hajeong Lee

**Affiliations:** 1 Department of Internal Medicine, Seoul National University Hospital, 101 Daehak-ro, Jongno-gu, Seoul, 03080 Korea; 2 Department of Biomedical Sciences, Seoul National University Graduate School, Seoul, Korea; 3 Department of Internal Medicine, Seoul National University College of Medicine, Seoul, Korea; 4 Department of Biomedical Sciences, Seoul National University College of Medicine, Seoul, Korea; 5 Department of Pathology, Seoul National University Hospital, Seoul, Korea; 6 Department of Pathology, Seoul National University College of Medicine, 101 Daehak-ro, Jongno-gu, Seoul, 03080 Korea

**Keywords:** Medical research, Kidney diseases

## Abstract

Mesangial proliferation is a diagnostic feature and a prognostic predictor of immunoglobulin A nephropathy (IgAN). We aimed to investigate the gene expression profiles of IgAN glomerulus according to the presence of mesangial proliferation. We performed spatial-specific transcriptomic profiling on kidney biopsy tissues using the GeoMx Digital Spatial Profiler. Twelve cases with three glomeruli for each case were profiled using direct pathologic classification (4 M1-IgAN, 4 M0-IgAN, and 4 donor controls). The results of enriched glom-specific genes demonstrated that M1-IgAN could be distinguished from controls (77 upregulated and 55 downregulated DEGs), while some DEGs were identified between M1-IgAN and M0-IgAN cases (24 upregulated and 8 downregulated DEGs) or between M0 and controls (1 upregulated and 16 downregulated DEGs). TCF21, an early podocyte damage marker, was the only differentially expressed gene (DEG) consistently upregulated in both M1-IgAN and M0-IgAN patients, whereas ATF3, EGR1, DUSP1, FOS, JUNB, KLF2, NR4A1, RHOB, and ZFP36 were consistently downregulated in IgAN cases. Glomeruli from M1-IgAN cases were significantly enriched for cell surface/adhesion molecules and gene expressions associated with vascular development or the extracellular matrix. Spatial transcriptomic analysis may contribute to dissecting structure-specific pathophysiology and molecular changes in IgAN.

## Introduction

Immunoglobulin A nephropathy (IgAN) is one of the most prevalent primary glomerular diseases in adults^1^. IgA nephropathy is a significant contributor to the medical and socioeconomic burden associated with kidney dysfunction, as approximately 20% of the patients’ progress to end-stage kidney disease^2^. Most of patients show initial kidney dysfunction, high blood pressure, proteinuria, or advanced histologic. However, patients without recognized clinical or pathological risk factors often experience benign IgAN. Such heterogeneity in clinical prognosis has led to research focusing on prognostic prediction and stratification of IgAN^3^, as treatment strategies may differ based on the risk profiles of individuals.

The most prevalent clinical feature of IgAN include acute glomerulonephritis and asymptomatic urinary abnormalities, such as microscopic hematuria or proteinuria. A significant proportion of asymptomatic IgAN patients are being detected due to frequent urine monitoring in schools and health screenings. This allows the early diagnosis of more IgAN patients with preserved kidney function. Consequently, pathologic characteristics play a significant role in both the diagnosis and risk stratification of IgAN. After the pathologic diagnosis is confirmed by glomerular IgA disposition and mesangial changes, the Oxford classification is widely used to sub-classify the pathologic findings of IgAN to predict long-term prognosis^4^.

Since the pathological features of IgAN reflect its mechanism and severity, transcriptome profiling targeting a specific pathologic alteration may reveal a potential therapeutic or prognostic biomarker related to IgAN pathophysiology^5,6^. In particular, mRNA profiles associated with mesangial proliferation in IgAN glomerulus may reflect the underlying mechanism of early disease development, as the glomerular mesangium is a key region where pathogenetic galactose-deficient IgA1 accumulates, and the inflammatory process initiates. Recent spatial transcriptomic analysis tools in nephrology revealed glom- or tubule-specific alterations in several kidney diseases^7,8^.

This study aimed to analyze the transcriptome profile of IgAN glomeruli using spatial transcriptomic analysis. We dissected individuals based on the extent of their mesangial proliferation and analyzed the pathology data to unravel transcriptome alterations specific to mesangial proliferation. We hypothesized that the glomerular IgAN transcriptome would reveal a panel of early transcriptomic biomarkers for the disease.

## Methods

### Ethical considerations

The Institutional Review Board of Seoul National University Hospital approved this investigation (IRB No. H-2205-104-1325). Informed consent was obtained from the patients before collecting any bio-specimens or clinical data. This study was performed in accordance with the Declaration of Helsinki.

### Study participants—IgAN cases

After obtaining informed consent, kidney biopsies were conducted at the study hospital, and the remaining tissue specimens were embedded in formalin-fixed paraffin-embedded (FFPE) blocks for future research or look-back diagnosis. The study population comprised IgAN patients from whom sufficient FFPE tissue blocks were available for initial diagnostic biopsies and provided informed consent for the use of the specimens for research purpose. The criteria were established to include IgAN appropriate for spatial transcriptomic analysis in relatively early development courses without severe kidney dysfunction, including cases (1) with a sufficient number of glomeruli (> 10 per section), (2) a recent biopsy (≥ year 2018), (3) no history of immunosuppressive treatment, (4) without comorbid diabetes mellitus, (5) with preserved estimated glomerular filtration rate (eGFR, ≥ 60 mL/min/1.73 m^2^), (6) and with established Oxford classification^4^. We also limited the age range from 20 to 59 years to exclude elderly individuals to minimize the aging effect on glomerular changes. Lastly, cases with cellular or fibro-cellular crescents were not included since an active immune status may imply fundamentally distinct characteristics and we also minimized the inclusion of cases with tubular atrophy/interstitial fibrosis or endocapillary proliferation^4,9^. Although some cases with segmental sclerosis or other glomerular lesions besides mesangial proliferation were included, we selected the target area of interest (AOI) for the spatial transcriptomic analysis based on the absence of these characteristics in the glomerulus.

Finally, biopsy specimens were analyzed by a pathologist to evaluate the degree of mesangial proliferation. Four cases of IgAN with notable mesangial proliferation (M1) and mild alterations (M0) were selected for each group. The pathologist marked the glomeruli with certain M1 and M0 findings for spatial transcriptomic analysis.

### Study participants—control group

Four donor kidney biopsy samples were included in the control group. Immediately after implantation, donor kidney biopsies were collected during the transplantation surgery. We selected the cases where the biopsies were performed in the same period as the IgAN cases (from 2018 to 2021). We included samples of all age groups (1 case each for the 20 s, 30 s, 40 s, and 50 s) and genders (2 male and 2 female cases). Donors were included if their urinalysis and blood tests revealed no evidence of kidney impairment (eGFR ≥ 80 mL/min/1.73 m^2^), hematuria, or proteinuria. In addition, pathological diagnosis, including Banff classification 2017, ensured that the donor control cases were glomerulopathy-free^10^.

### Spatial transcriptomic analysis

We prepared the slides according to the manufacturer’s guidelines (NanoString GeoMx DSP Manual Slide Preparation; MAN-10150-01). To avoid batch effects, three slides, each comprising four biopsy specimens from all three groups, were uploaded. For FFPE blocks, 5 μm-thick tissue sections were mounted on charged slides (Leica BOND Plus slides) to ensure that they fit within the scanned area of the slide. Slides were baked at 60 ℃ for 30 min, deparaffinized using CitriSolv, and rehydrated using ethanol and 1× phosphate-buffered saline. Target epitopes were retrieved and exposed by incubating in 1× Tris–EDTA, pH 9, and preheated 1 μg/ml proteinase K, respectively. After washing in 1× PBS, the slides were incubated overnight at 37 ℃ with RNA detection probes linked to the DNA oligo barcode with a UV photocleavable linker for NGS readout WTA (Whole Transcriptome Atlas). The following day, slides were stained with SYTO 13 (NanoString, 121300303), CD68 (Novus, NBP2-34587 AF532), PanCK (Novus, NBP2-33200 AF594), and alpha Smooth Muscle Actin (Abcam, ab202296) for 1 h at room temperature, and then loaded into the GeoMx DSP instrument for scanning and selecting the AOI. Oligonucleotides encoding their target genes were cleaved and released from the selected AOI, and an average of 173.3125 cells/AOI were collected from the DSP collection plate. During polymerase chain reaction (PCR), primer pairs and i5 and i7 dual-indexing sequences were used to amplify and index the oligonucleotides. The libraries were pooled into a single microtube and cleaned using AMPure beads. Using the Agilent 4200 TapeStation system, the libraries appeared around 174 base pairs and were sequenced on the Illumina Counting Platform, NextSeq 6000, with 27 × 27 paired-end reads.

### Bioinformatics and statistical analysis

Raw fastq files were converted into digital count conversion files using the GeoMx NGS Pipeline software (v2.0). We used the DSP Data Analysis Suite (v2.4) and quality-controlled results. Gene expression lower than the limit of quantitation 2 were initially disregarded. The limit of quantitation was set as the negative probe geomean multiplied by the geometric standard deviations of the negative probes. In addition, genes that were not expressed in more than half of the included samples were excluded from the downstream analysis.

Unnormalized read counts of the filtered genes were used to draw relative log expression plots to identify technical outliers. Principal component analysis was used to plot the similarities and differences between the read counts of the included AOIs.

To identify differentially expressed genes (DEGs), we used the DESeq2 package in R (version 3.6.2)^11^. We compared the differences between the AOIs group IgAN and controls, and between the M0, M1, and control groups. Genes with a false-discovery rate < 0.1 by the Benjamini–Hochberg correction, were considered DEGs. The identified DEGs were analyzed using the Toppgene suite mainly for gene ontology annotation^12^. To investigate the protein–protein interaction network of the identified DEGs, we used the STRING database and the DEGs with at least one significant connection with another DEG were presented^13^.

### Immunohistochemistry staining

We validated the target biomarker, TCF21 and THY1, by additional immunohistochemistry staining in protein level. We included total of 12 IgAN cases and 4 controls. The slides were placed in a 60 ℃ oven for 30 min and subsequently immersed in xylene to eliminate paraffin. Tissue samples underwent hydration through a series of ethanol concentrations (100%, 90%, 80%, 70%, 50%) and distilled water (DW). Antigen retrieval was achieved by heating with EDTA (pH 8.0) at 98 ℃ for 20 min, followed by a 5-min wash with with DW. Hydrophobic barrier was established around the tissue sections. Hydrogen peroxide (H_2 O_2) was dispersed onto the tissues to cover the entire section. Tissue samples were then incubated with 2% bovine serum albumin (BSA) for 1 h. Subsequently, tissue sections were probed overnight at 4 ℃ in a humidified chamber with the following primary antibodies: anti-TCF21 (MBS9612236; MyBioSource, dilution 1:100), and anti-Thy1 (ab92574; abcam, dilution 1:50, [EPR3132]). On the subsequent day, tissue samples were subjected to a 30-min incubation with secondary antibodies at room temperature (RT), followed by two washes with 1× tris-buffered saline, 0.1% tween 20 (1× TBST). The secondary antibody used was anti-rabbit AP-conjugated antibody (18653S, Cell Signaling Technology). A substrate mixture was applied to all slides, and the tissues were counterstained with hematoxylin and sodium bicarbonate for 5 s and 30 s, respectively. Immunohistochemistry (IHC) images were acquired using an Axioscan 7 microscope slide scanner (ZEISS, Germany). We quantified the staining proportion in 3 glomeruli in each case using the Image J. An automated macro script was used for quantification to reduce measurement bias.

## Results

### Characteristics of the study participants

The characteristics of the study participants are summarized in Table 1. IgAN patients ranged in age from 20 to 54 years. None of the patients had a history of diabetes or consumed immunosuppressive drugs. With relatively large numbers of glomeruli included in their biopsies, one IgAN case with M1 finding had E1, and four IgAN cases (one from M0 and three from the M1 group) had segmental sclerosis. All IgAN cases had eGFR > 60 mL/min/1.73 m^2^ and those with M1 findings had higher baseline proteinuria. All IgAN cases with M1 glomerulus exhibited podocyte foot process effacements, which were present in 2 IgAN cases with M0 glomerulus, and two control cases. Regarding the zero-time allograft biopsy controls, none of the cases had significant pathological changes in the glomerulus, and only one case had low-grade changes in tubular atrophy and interstitial fibrosis. No significant proteinuria was observed in the control group.

**Table 1:** Baseline characteristics of the study subjects.

Group	Age	Sex	RAASB	ISD	Pathologic classification	Glom N	Global sclerosis N (%)	Segmental sclerosis N (%)	Crescent N (%)	Diabetes	Hypertension	eGFR (mL/min/1.73 m^2^)	proteinuria (g/g or g/24 h)	Electron microscopy		
														Electron-dense deposit	GBM	Foot process effacement
M0	30s	F	(−)	(−)	M0, E0, S0, T0, C0	12	0 (0%)	0 (0%)	0 (0%)	(−)	(−)	110.3	0.34	Small mesangial	(−)	Focal slight
M0	50s	F	(+)	(−)	M0, E0, S1, T1, C0	25	2 (8%)	1 (4%)	0 (0%)	(−)	(+)	86.9	0.51	Small mesangial	(−)	(−)
M0	30s	M	(−)	(−)	M0, E0, S0, T0, C0	25	0 (0%)	0 (0%)	0 (0%)	(−)	(−)	110.3	0.14	Small mesangial	(−)	Focal mild
M0	20s	M	(−)	(−)	M0, E0, S0, T0, C0	27	0 (0%)	0 (0%)	0 (0%)	(−)	(−)	134.70	0.18	Small mesangial	(−)	(−)
M1	30s	F	(−)	(−)	M1, E0, S1, T0, C0	15	2 (13%)	3 (20%)	0 (0%)	(−)	(−)	105.8	2.44	Small mesangial	(−)	Focal mild
M1	20s	F	(−)	(−)	M1, E0, S1, T0, C0	59	8 (14%)	2 (3%)	0 (0%)	(−)	(−)	136.2	3.13	Moderate mesangial	(−)	Focal slight
M1	50s	M	(+)	(−)	M1, E0, S1, T0, C0	34	7 (21%)	4 (12%)	0 (0%)	(−)	(+)	67.50	1.66	Small mesangial	Thickening	Focal mild
M1	30s	F	(+)	(−)	M1, E1, S0, T0, C0	43	0 (0%)	0 (0%)	0 (0%)	(−)	(−)	120.60	1.81	Moderate mesangial, small subendothelial	(−)	Focal moderate
Control	20s	M	(−)	(−)	Banff: no positive findings	75	0 (0%)	0 (0%)	0 (0%)	(−)	(−)	112.6	Not detected	Small mesangial	(−)	Focal mild
Control	30s	F	(−)	(−)	Banff: no positive findings	76	8 (11%)	0 (0%)	0 (0%)	(−)	(−)	113.2	Not detected	(−)	(−)	Focal slight
Control	40s	M	(−)	(−)	Banff: no positive findings	24	0 (0%)	0 (0%)	0 (0%)	(−)	(−)	111.9	Not detected	(−)	(−)	(−)
Control	50s	F	(−)	(−)	Banff: interstitial fibrosis/tubular atrophy grade 1 (ci1, ct1, i-IFTA2, t-IFTA1)	42	3 (7%)	0 (0%)	0 (0%)	(−)	(−)	90.2	Not detected	(−)	(−)	(−)

RAASB, renin–angiotensin–aldosterone system blockade; ISD, immunosuppressive drugs; eGFR, estimated glomerular filtration rate; GBM, glomerular basement membrane.

**Figure 1:**
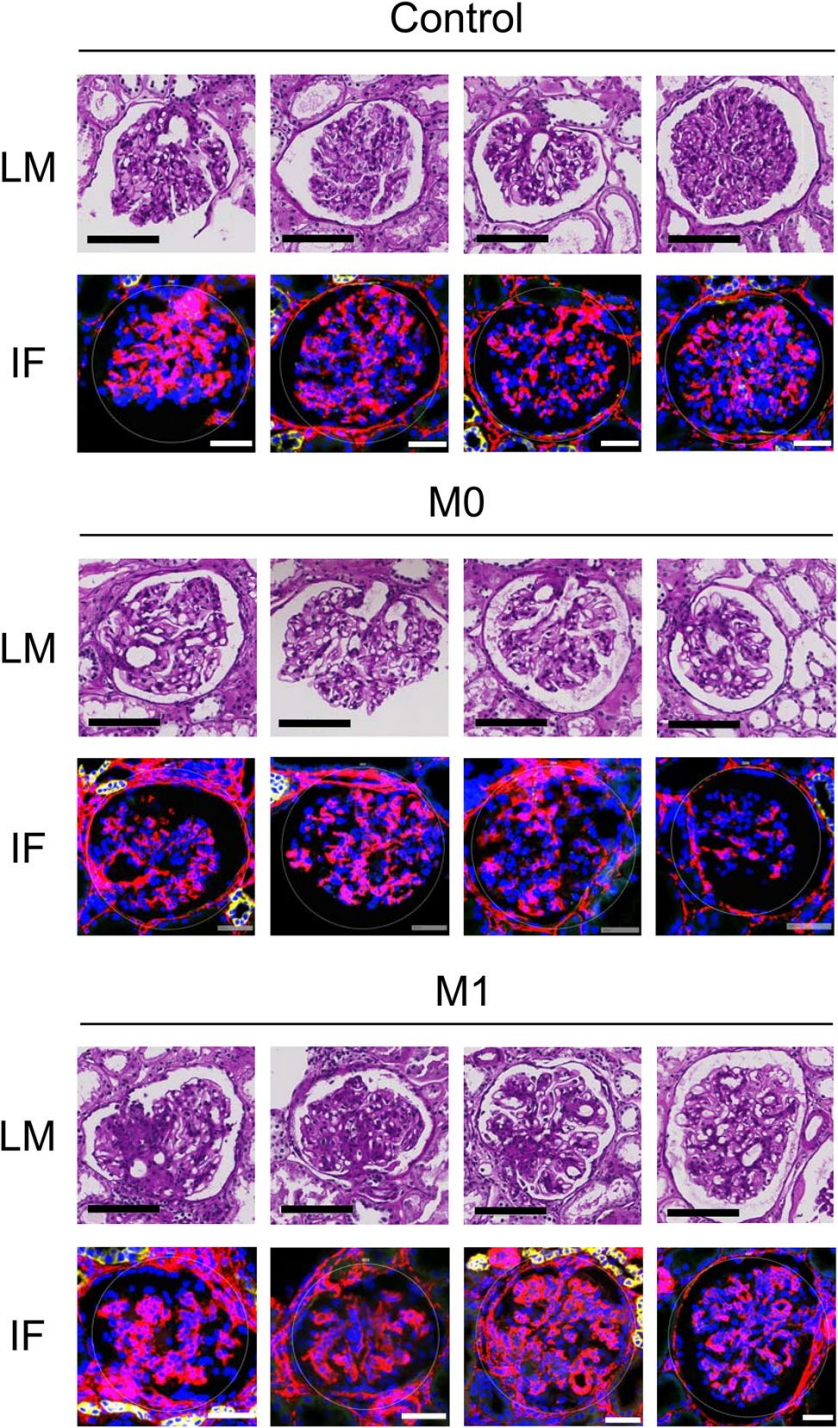
Representative images of the targeted glomerulus in the study. The light microscopy (bars = 100 µm) and immunofluorescence images (blue, SYTO13; yellow, panCK; red, alpha Smooth Muscle Actin; green, CD68) are shown in the left and right graphs, respectively. A pathologist annotated the degree of mesangial proliferation in each glomerulus as part of a spatial transcriptome profiling study. The scale bars indicate 100 μm for light microscopy images and 50 μm for immunofluorescence images.

Representative light microscope biopsy slide scan results with periodic acid-Schiff (PAS) staining are presented in Fig. 1. We carefully selected the target AOI glomerulus to include significant and mild mesangial alterations in the M1 group and M0 groups, respectively. The results for the controls were typical.

### Spatial transcriptomic profiling results

Round AOIs with a median of 205.8 (interquartile ranges 192.0, 222.9) µm were drawn to include a median of 185 (139, 211) cells per AOIs (Fig. 1). A median of 1857156 reads was aligned from the RNA-seq, with all cases reaching > 80% of Q30 bases and a median mean quality score of 35 (interquartile range 34.9, 35.4). After we filtered the genes with expression levels below the limit of quantitation or prevalence below 50% of the AOIs, 14888 genes remained for the downstream analysis.

**Figure 2:**
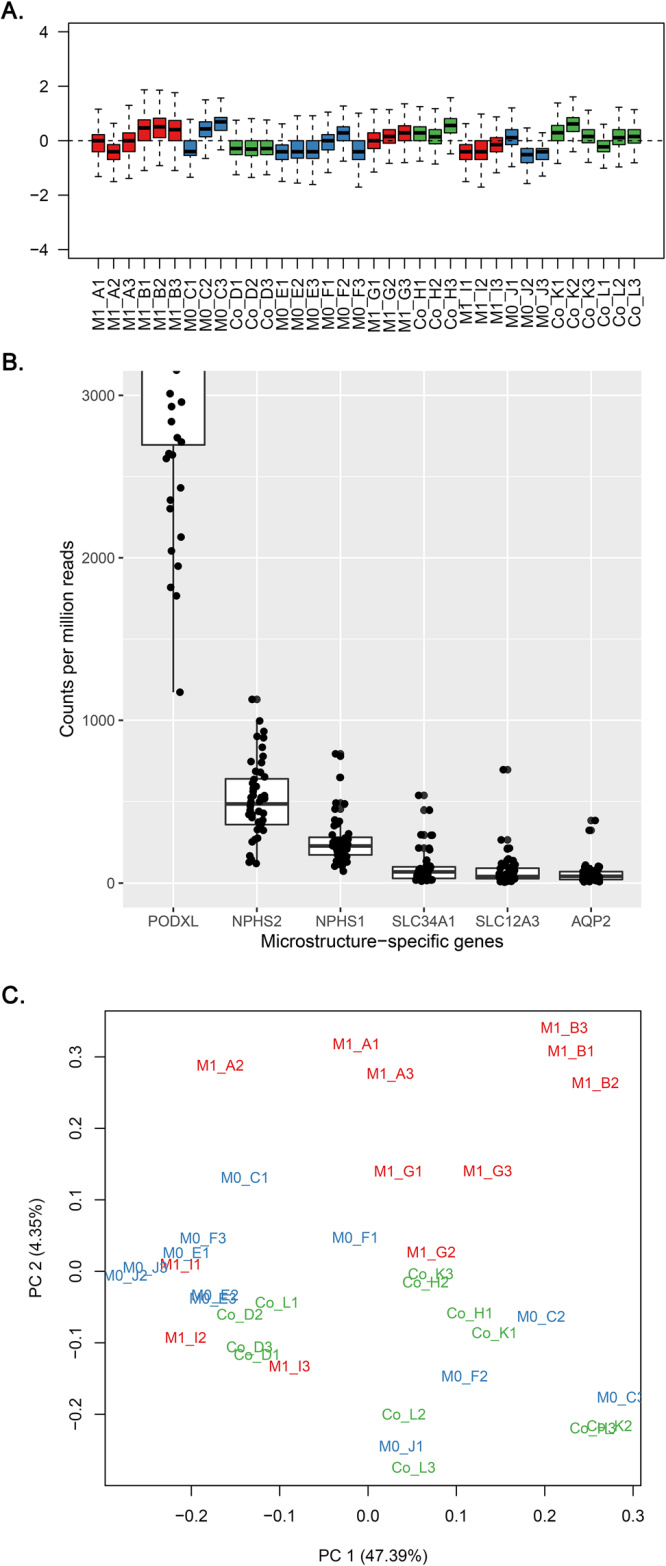
The initial quality control process of the read count results. (**A**) Relative log expression plot revealed no notable batch effects. The read counts from each area of interest are shown using box plots in the same order as the samples mounted on the experiment slides. Each slide included 12 area-of-interests from four cases with three glomeruli each (red, M1 cases; blue, M0 cases; green, control donor-kidney). (**B**) Kidney microstructure-specific gene expression profiles indicated a relative enrichment of glomerulus-specific genes (PODXL, NPHS2, and NPHS1) when compared to proximal convoluted tubule (SLC34A1), distal convoluted tubule (SLC12A3), and collecting duct (AQP2)-specific genes. To show the differences between other gene expression amounts, PODXL box plot was cropped and the glom-specific gene expression was evidently abundant. (**C**) Principal component analysis showed certain differences between the study groups (red, M1 cases; blue, M0 cases; green, control donor-kidney), which was particularly prominent for the M1 cases.

Relative log expression plots supported the low likelihood of extreme outliers (Fig. 2A). When gene expression profiles for kidney substructure-specific genes were initially analyzed, the counts per million expression ranks for well-known glomerulus-specific genes were significantly higher (PODXL, rank 4; NPHS2, rank 108; NPHS1, rank 331) than those for proximal convoluted tubule (SLC34A1, rank 3005), distal convoluted tubule (SLC12A3, rank 2402), or collecting duct (AQP2, rank 2878) (Fig. 2B)^14^. The findings supported that the GeoMx DSP successfully captured the enriched genes within the glomerulus, despite the large amounts of tubulointerstitial tissue in kidney biopsy samples.

The principal component analysis results revealed some overlap between the M0, M1, and control AOIs, as well as the certain discrimination for the M1 group (Fig. 2C).

### Differentially expressed genes between IgAN and controls

**Figure 3:**
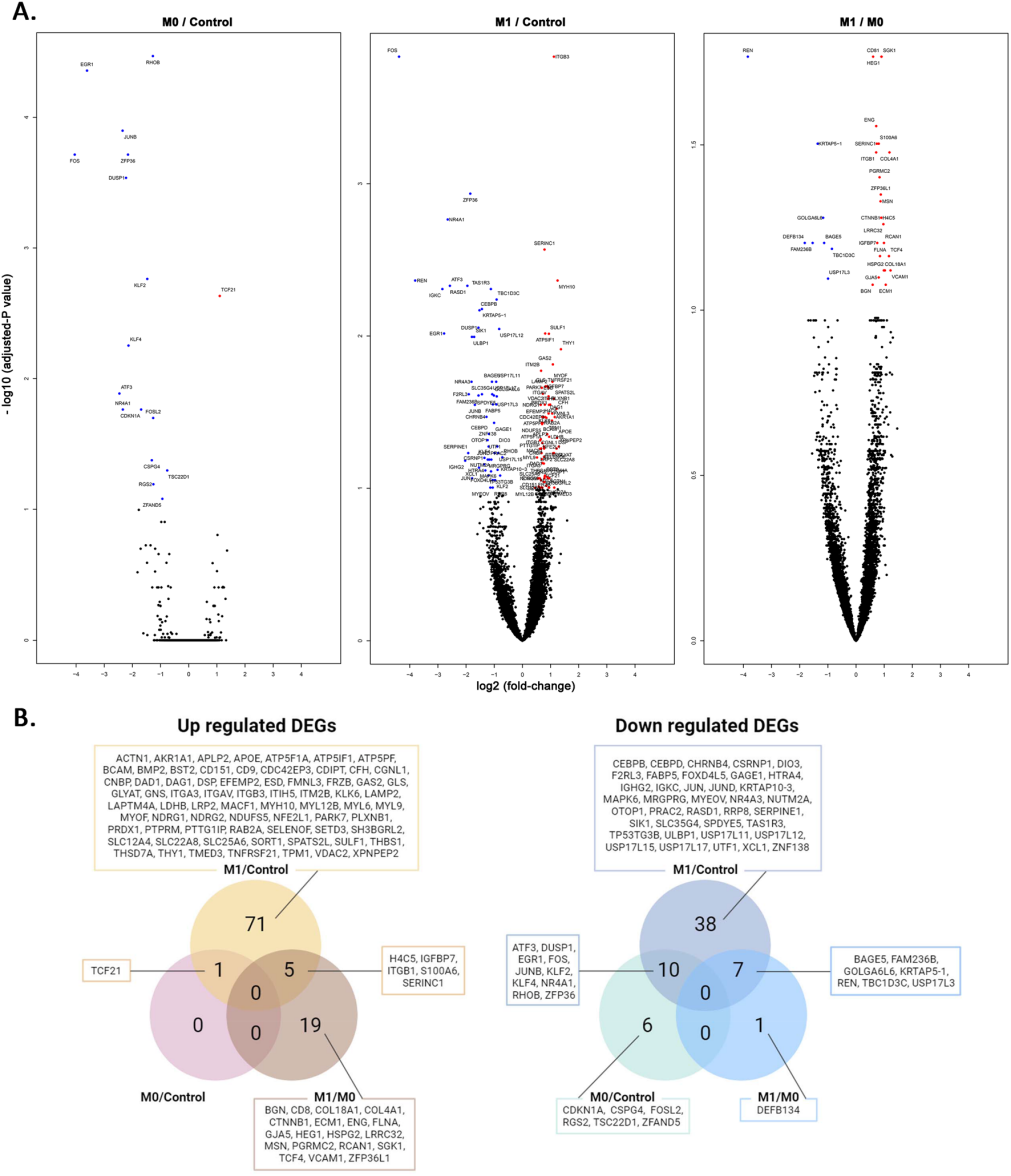
The differentially expressed genes (DEGs). (**A**) Volcano plots. X-axes indicate log2 (fold-change), and Y-axes indicate − log10 (adjusted-P value). Significantly (adjusted-P value < 0.1) downregulated and upregulated genes are marked in blue and red, respectively. DEGs between IgAN M0 cases and controls (left), IgAN M0 cases and controls (center), and IgAN M1 and IgAN M0 cases (right). (**B**) Venn-diagram showing the overlapping or specific DEGs in each target group.

**Figure 4:**
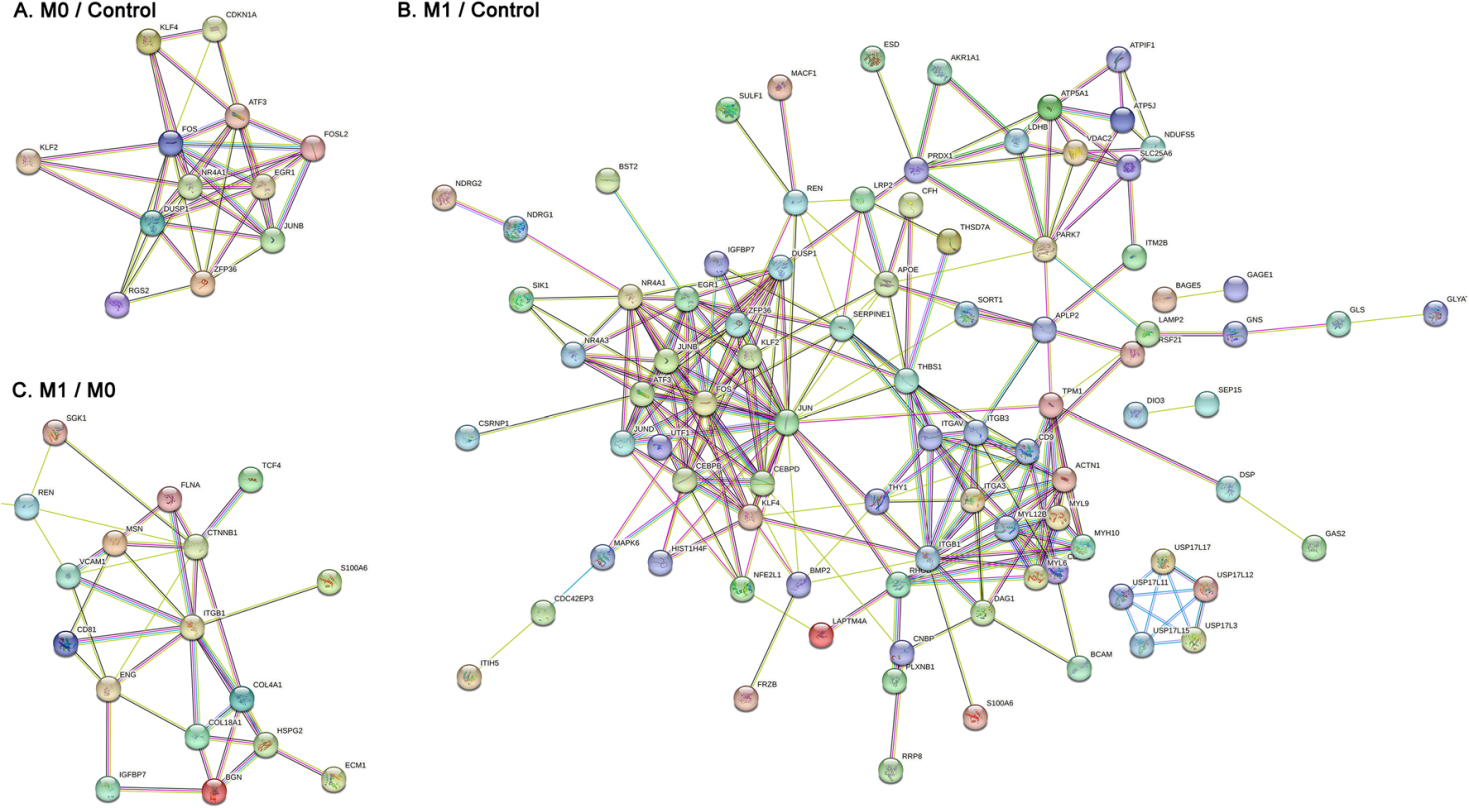
Protein–protein interaction network analysis. (**A**) IgAN M0 cases and controls, (**B**) IgAN M1 cases and controls, and (**C**) IgAN M1 cases and M0 cases were compared using the STRING database, and the DEGs with at least one significant relationship with another DEG are displayed in each comparison.

A compilation of all genes whose expression levels differed significantly across groups is presented in Supplemental Table 1. Using false-discovery rate limits of 0.1, we identified 16 downregulated DEGs between M0 and control, and one upregulated DEG (transcription factor 21, TCF21) (Table 2, Fig. 3A and Fig. 3B). Five of 16 downregulated DEGs between M0 and controls were annotated with a GO term related to DNA-binding transcriptional activator activity (Table 3 and Supplemental Table 2). The genes included in the GO term were Fos proto-oncogene (FOS), KLF transcription factor 2 (KLF2), KLF4, early growth response 1 (EGR1), and JunB proto-oncogene (JUNB). FOS and JUNB were annotated as the transcription factor AP-1 complex. Regarding the biological process GO term, seven downregulated genes were annotated to the cellular response to endogenous stimuli, including cytokine responses, hormonal stimuli, and oxidative stress. Analysis of protein–protein interactions revealed that the JUNB/FOS-related molecules interacted with one another (Fig. 4A).

**Table 2:** List of identified DEGs between the study groups.

Groups	Directions	DEGs
M0/Control	Up	TCF21
	Down	ATF3, CDKN1A, CSPG4, DUSP1, EGR1, FOS, FOSL2, JUNB, KLF2, KLF4, NR4A1, RGS2, RHOB, TSC22D1, ZFAND5, ZFP36
M1/M0	Up	BGN, CD81, COL18A1, COL4A1, CTNNB1, ECM1, ENG, FLNA, GJA5, H4C5, HEG1, HSPG2, IGFBP7, ITGB1, LRRC32, MSN, PGRMC2, RCAN1, S100A6, SERINC1, SGK1, TCF4, VCAM1, ZFP36L1
	Down	BAGE5, DEFB134, FAM236B, GOLGA6L6, KRTAP5-1, REN, TBC1D3C, USP17L3
M1/Control	Up	ACTN1, AKR1A1, APLP2, APOE, ATP5F1A, ATP5IF1, ATP5PF, BCAM, BMP2, BST2, CD151, CD9, CDC42EP3, CDIPT, CFH, CGNL1, CNBP, DAD1, DAG1, DSP, EFEMP2, ESD, FMNL3, FRZB, GAS2, GLS, GLYAT, GNS, H4C5, IGFBP7, ITGA3, ITGAV, ITGB1, ITGB3, ITIH5, ITM2B, KLK6, LAMP2, LAPTM4A, LDHB, LRP2, MACF1, MYH10, MYL12B, MYL6, MYL9, MYOF, NDRG1, NDRG2, NDUFS5, NFE2L1, PARK7, PLXNB1, PRDX1, PTPRM, PTTG1IP, RAB2A, S100A6, SELENOF, SERINC1, SETD3, SH3BGRL2, SLC12A4, SLC22A8, SLC25A6, SORT1, SPATS2L, SULF1, TCF21, THBS1, THSD7A, THY1, TMED3, TNFRSF21, TPM1, VDAC2, XPNPEP2,
	Down	ATF3, BAGE5, CEBPB, CEBPD, CHRNB4, CSRNP1, DIO3, DUSP1, EGR1, F2RL3, FABP5, FAM236B, FOS, FOXD4L5, GAGE1, GOLGA6L6, HTRA4, IGHG2, IGKC, JUN, JUNB, JUND, KLF2, KLF4, KRTAP10-3, KRTAP5-1, MAPK6, MRGPRG, MYEOV, NR4A1, NR4A3, NUTM2A, OTOP1, PRAC2, RASD1, REN, RHOB, RRP8, SERPINE1, SIK1, SLC35G4, SPDYE5, TAS1R3, TBC1D3C, TP53TG3B, ULBP1, USP17L11, USP17L12, USP17L15, USP17L17, USP17L3, UTF1, XCL1, ZFP36, ZNF138

DEGs with false discovery rate < 0.1 are presented in the table.

**Table 3:** Top gene ontologies in each annotation results.

Comparison	Direction	Domain	Gene ontology terms	False discovery rate	Annotated gene lists
M0/Control	Up	Biological process	GO:0072275 (metanephric glomerulus morphogenesis)	0.004	TCF21
		Molecular function	GO:0050681 (nuclear androgen receptor binding)	0.019	TCF21
		Cellular component	NA	NA	NA
	Down	Biological process	GO:0034097 (response to cytokine)	< 0.001	FOS, ZFP36, KLF4, DUSP1, EGR1, KLF2, JUNB
		Molecular function	GO:0000978 (RNA polymerase II cis-regulatory region sequence-specific DNA binding)	< 0.001	FOS, KLF4, EGR1, KLF2, JUNB
		Cellular component	GO:0000785 (chromatin)	0.002	FOS, KLF4, EGR1, KLF2, JUNB
M1/Control	Up	Biological process	GO:0048646 (anatomical structure formation involved in morphogenesis)	< 0.001	TPM1, CFH, FMNL3, CNBP, BMP2, MYOF, THBS1, MYH10, ATP5IF1, THY1, MYL9, THSD7A, TCF21, CD9, IGFBP7, PTPRM, SULF1, CDIPT, LRP2, DAG1, MACF1, ACTN1, ITGA3, ITGAV, ITGB1, ITGB3, TNFRSF21
		Molecular function	GO:0050839 (cell adhesion)	< 0.001	THBS1, NDRG1, THY1, CD9, PTPRM, DSP, PARK7, PRDX1, MACF1, CD151, ACTN1, BCAM, ITGA3, ITGAV, ITGB1, ITGB3
		Cellular component	GO:0009986 (cell surface)	< 0.001	SORT1, CFH, ATP5PF, BMP2, THBS1, ATP5IF1, THY1, CD9, BST2, SULF1, LRP2, DAG1, CD151, BCAM, ITGA3, APOE, ITGAV, ITGB1, ITGB3, ATP5F1A
	Down	Biological process	GO:0033993 (response to lipid)	0.002	EGR1, FOS, ZFP36, DUSP1, REN, CEBPB, NR4A1
		Molecular function	GO:0001216 (DNA-binding transcription activator activity)	< 0.001	EGR1, FOS, ATF3, CEBPB, NR4A1
		Cellular component	GO:0005667 (transcription regulator complex)	0.015	FOS, ATF3, CEBPB, NR4A1
M1/M0	Up	Biological process	GO:0001944 (vascular development)	< 0.001	COL4A1, HSPG2, FLNA, COL18A1, TCF4, GJA5, HEG1, CTNNB1, IGFBP7, RCAN1, ZFP36L1, ECM1, ENG, ITGB1, VCAM1, MSN
		Molecular function	GO:0005201 (extracellular matrix constituent)	< 0.001	COL4A1, HSPG2, COL18A1, IGFBP7, ECM1, ENG, BGN
		Cellular component	GO:0030312 (extracellular matrix)	< 0.001	COL4A1, S100A6, HSPG2, COL18A1, IGFBP7, ECM1, ITGB1, LRRC32, BGN
	Down	Biological process	NA	NA	NA
		Molecular function	NA	NA	NA
		Cellular component	NA	NA	NA

The gene ontology terms with the lowest false discovery rate in each annotation analysis are presented in table.

The marked increase in DEGs between M1 and the control group is indicative of the significant difference in glomerulus between the two groups. We identified 77 upregulated, and 14 downregulated DEGs (Table 2, Fig. 3A and Fig. 3B). TCF21 was included in the significantly upregulated DEGs when comparing M0 to the control group. The consistently down-regulated DEGs in M1 and M0 IgAN were FOS, DUSP1, EGR1, and ZFP36. When we annotated the DEGs identified between M1 and controls, DNA-binding transcription factor activity was the most frequently reported molecular function GO term annotated by downregulated genes, and four genes were annotated to transcription regulator complex cellular components (Table 3). Next, we annotated the 77 upregulated DEGs identified in M1 relative to controls; the most frequently annotated molecular function domain was cell adhesion molecule binding, extracellular matrix binding, or integrin binding-related GO terms. Regarding the biological process GO terms, cell adhesion, endothelial cell proliferation, and cell–matrix adhesion were notable domains with significantly enriched results. Functional network analysis also reported similar findings, as the JUN/FOS-related molecules were clustered, while the integrin- or myosin-related molecules were strongly interconnected (Fig. 4B). The ubiquitin-specific peptidase 17-like family member (USP17LX) cluster was also identified among the upregulated DEGs.

### Differentially expressed genes between M1 and M0 IgAN

We identified 24 upregulated and 8 downregulated DEGs between the M1 and M0 glomerulus (Table 2, Fig. 3A, B, and Supplemental Table 1). When the DEGs were annotated (Table 3 and Supplemental Table 2), a total of 7/24 upregulated DEGs were annotated to extracellular matrix structural constituents (GO:0005201), including collagen alpha-1(IV) chain (COL4A1), COL18A1, heparin sulfate proteoglycan 2 (HSPG2), insulin-like growth factor binding protein 7 (IGFBP7), extracellular matrix protein 1 (ECM1), endoglin (ENG), and biglycan (BGN) (Table 3). In addition, cell adhesion molecule binding (GO:0050839) molecular function GO terms were another notable GO term, with 7/24 upregulated DEGs annotated. In addition, nine genes were annotated to signal receptor binding (GO:0005102) molecular function GO terms, including HSPG2, ECM1, ENG, filamin A (FLNA), CD81, catenin beta 1 (CTNNB1), integrin subunit beta 1 (ITGB1), vascular cell adhesion molecule 1 (VCAM1), and moesin (MSN). For biological process GO terms, most of the upregulated DEGs (up to 16/24) were annotated to vascular development-related GO terms, such as collagens (e.g., COL4A1, COL18A1) or cell adhesion-related molecules (e.g., ITGB1, VCAM1). The most commonly annotated cellular component GO terms were related to the plasma membrane, including the integral component of the plasma membrane (GO:0005887), membrane protein complex (GO:0098796), intrinsic component of the plasma membrane (GO:0031226), and plasma membrane region (GO:0098590). The functional network analysis clustered cell adhesion molecules and extracellular matrix components (Fig. 4C).

### Immunohistochemistry analysis results

**Figure 5:**
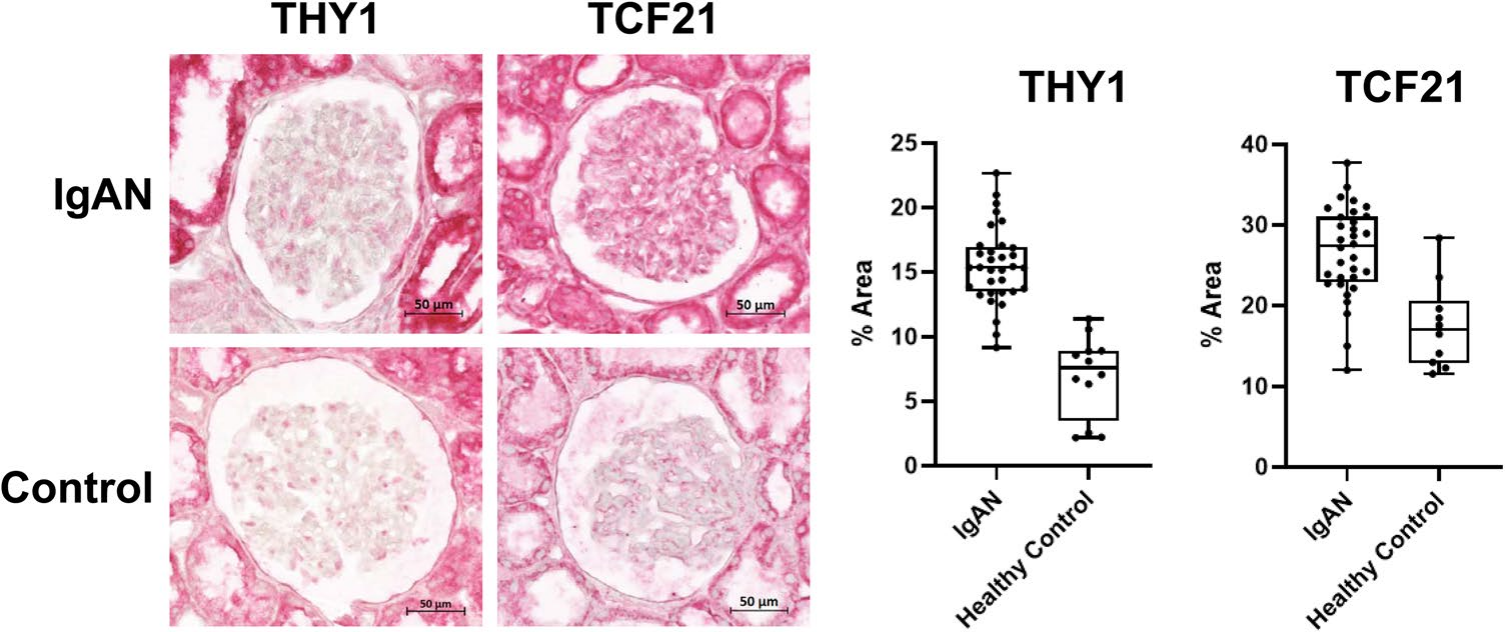
Immunohistochemistry results for validation in protein level for TCF21 and THY1. Total of 12 case of IgAN and 4 healthy controls are included in the staining and 3 glomeruli in each case were selected for quantification. Representative images are shown in the figure. Quantification was performed by image J using an automated macro script. The quantified abundance of protein expression was compared in the according box plots.

In our immunohistochemistry staining for validation purpose in protein-level, TCF21, a representative DEG reflecting podocyte injury, was more abundant within IgAN glomerulus when compared to within those of the controls (Fig. 5). When we tested THY1, the expression of THY1 was significantly higher in IgAN glomerulus than control glomerulus, which was in consistency with our spatial transcriptomic results.

## Discussion

Using innovative spatial transcriptomic profiling, we identified DEGs that were altered in the IgAN glomeruli. Transcriptomic profiling revealed significant differences between M1 and M0 glomeruli regarding mesangial proliferation, although a frequently altered signature was also detected in early IgAN without significant mesangial proliferation. The current findings provide insight into the pathophysiological alterations during the development and progression of IgAN.

The pathophysiology of IgAN is explained by the glomerular accumulation of IgA1 and associated abnormal galactosylated immune complexes^15^. Such pathogenic deposition of IgA1 and immune complexes is a pathologic hallmark of IgAN and the consequent mesangial expansion of the glomerulus. The degree of mesangial proliferation reflects the disease severity of IgAN; those with M1 show a worse prognosis than M0^4,16^.

The current literature suggests that mesangial expansion and related inflammatory signals induce pathologic alterations in the tubulointerstitial tissue of IgAN^17,18^. Together, disrupted glomerular and tubular structures lead to proteinuria, a distinctive clinical indicator of disease activity^3^, and may cause non-reversible impairment of kidney function. IgAN patients having glomeruli with IgAN-specific pathologic alterations may represent the early pathophysiological mechanism of IgAN in the absence of clinical risk factors for progression. Thus, attempts have been made to characterize gene expression profiles in the glomeruli of clinically early IgAN patients^5,6^. However, due to the difficulty in identifying the gene expression profiles of the glomerulus with directly annotated pathological readings, transcriptomic profiles of IgAN glomerulus concerning the degree of mesangial expansion have not yet been performed. In this study, IgAN transcriptome profiles were evaluated in relation to the degree of mesangial changes using a recently introduced spatial transcriptomic profiling strategy in the field of nephrology^7^. Our study has the following strengths: We (1) profiled the transcriptome of IgAN glomeruli based on pathology annotations; (2) included early IgAN patients without established reduction in eGFR to reflect the early pathophysiologic signatures; (3) included allograft implantation biopsy controls, which could have certain power as clean-controls compared to other types of control tissues commonly used in the field (e.g., tumor nephrectomy tissues); and (4) the profiles successfully enriched the gene expression profiles from the glomerulus. Our study revealed that the IgAN glomeruli have diverse gene expression profiles according to the degree of mesangial proliferation.

When comparing M1 and M0 glomeruli to controls, TCF21 was the only upregulated DEG. TCF21 is a mesoderm-specific gene that plays a vital role in the embryonic development of glomerular epithelial cells in the kidney and is primarily expressed in podocytes. Previous studies demonstrated that lack of TCF21 impaired mesangial cell differentiation^19,20^. Moreover, TCF21 expression in podocytes was markedly elevated in injured glomeruli of IgAN, which was suggested to protect podocyte function after damage^21^. In a previous study, a correlation between the degree of proteinuria in IgAN patients and TCF21 protein expressions was also identified^22^. The significance of TCF21 in proteinuric glomerulonephritis and IgAN allows the detection of podocyte injury even in early IgAN cases without evident glomerular pathologic abnormalities.

Transcription factor AP-2-related FOS and JUN gene expression profiles were downregulated in IgAN glomeruli. Despite the pro-inflammatory function of the JUN/FOS pathway, its reductions have been frequently observed in IgAN. In our previous transcriptomic investigation of hand-microdissected IgAN glomeruli, these genes were also significantly reduced in IgAN patients^6^. The current findings are comparable with those of a previous microarray-based transcriptome profiling study of IgAN glomerulus, which also demonstrated a significant decrease in FOS, JUN, FOSB, EGR1, and DUSP1 in IgAN^23^. Therefore, the early downregulation of the JUN/FOS pathway may play a crucial role in IgAN pathophysiology, although the precise mechanism needs to be elucidated in future experimental studies.

Another notable gene that was downregulated in IgAN glomerulus was DUSP1. DUSP1, an inhibitor of the mitogen-activated protein kinase (MAPK) pathway, was considerably down-regulated in IgAN glomeruli and tubules, according to our previous transcriptome investigation^24^. Additional investigation is required to understand whether the upregulated MAPK pathway can be a specific treatment target to prevent progression of IgAN.

The transcriptomic profiles of the M1 glomeruli were markedly different from those of the control glomerulus, which is expected considering the disease severity. The increase of THY1, an indicator of mesangial proliferation^25^, gives credibility to the current findings. Since the extracellular matrix predominantly comprises the expanded mesangium, the extracellular matrix component-related molecules were likely the majority of upregulated genes in the M1 glomerulus.

The current profiles suggest that the process associated with endothelial proliferation may occur during the active phase of IgAN, and the expression of inflammatory cell adhesion molecules in IgAN glomerulus increases as mesangial proliferation progresses. Therefore, we infer that the current findings reflect the time-sequence of pathophysiological mechanism of IgAN according to the degree of mesangial proliferation.

Future applications of the current study results require further investigation of the therapeutic relevance of the reported DEG lists. Future studies may target TCF21, DUSP1, or JUN/FOS-related pathways to develop a medical strategy to block or reverse the pathophysiological development of IgAN. In addition, the transcriptome profiles of the M1 glomerulus have been updated to include mesangial proliferation-linked molecules; hence, these targets may be considered when assessing the severity of IgAN. A mechanistic investigation may also reveal a therapeutic biomarker for IgAN to reduce disease severity and mesangial proliferation.

Furthermore, we could see than 6 of the 10 DEGs downregulated in both M0 and M1 IgAN are also identified in our previous glom bulk-seq data of IgAN^6^. This support the validity of the current IgAN spatial transcriptomic analysis and the common genes repetitively identified from different technology should be prioritized in the future investigation of pathophysiologic mechanism of IgAN. The strength of the current study is that we could measure the amount of mRNA amounts from pathology-annotated glomerulus with clear dissection by the advanced spatial technology and that we could include donor kidney biopsy samples as controls. On the other hand, the potential weakness includes that the amount of glomerulus tissue from thin biopsy slide is small which might have resulted in low statistical power and read depth.

The current study had several limitations. First, the sequencing depth of the transcriptomic profiles was difficult to determine, considering the small sample size, limiting the sensitivity of the current study. For example, nominal changes in genes with relatively low absolute expression may not have been identified as significant DEG. Thus, besides the reported DEGs, the total list may need to be reviewed with a less stringent interpretation of the P-values, and the genes not reported as DEGs may still have clinical significance. Additional large scale study including various primary glomerulonephritis may be helpful to reveal the pathophysiologic mechanism of IgAN with a larger power. Second, the gene expression profiles might have been affected by AOI selection. Intra-individual variations in certain genes were prominent. This implies that spatial transcriptomic profiling of glomeruli with low mRNA levels may be easily modified by factors such as the inclusion of juxtaglomerular cells or tubular cells, although these modifications were likely unintended. Finally, the current study only included East Asian cases from a single university hospital; thus, future research should broaden the generalizability of the current findings.

In conclusion, spatial transcriptomic analysis of IgAN glomeruli facilitates profiling the gene expression signature based on the degree of mesangial proliferation. The findings and approaches may be used in future studies to better comprehend the pathophysiology of IgAN and to identify disease-specific biomarkers.

### Supplementary Information

Supplementary Table 1.

Supplementary Table 2.

## Data Availability

Data for this study is shared in a public repository, figshare (doi: 10.6084/m9.figshare.24906435). The individual data for identification of individual diagnosis information is available by the corresponding author upon reasonable request.
